# A veterinary perspective on One Health in the Arctic

**DOI:** 10.1186/s13028-017-0353-5

**Published:** 2017-12-16

**Authors:** Christian Sonne, Robert James Letcher, Bjørn Munro Jenssen, Jean-Pierre Desforges, Igor Eulaers, Emilie Andersen-Ranberg, Kim Gustavson, Bjarne Styrishave, Rune Dietz

**Affiliations:** 1 Department of Bioscience, Arctic Research Centre (ARC), Aarhus University, Faculty of Science and Technology, Frederiksborgvej 399, PO Box 358, 4000 Roskilde, Denmark; 2 Ecotoxicology and Wildlife Health Division, Environment and Climate Change Canada, National Wildlife Research Centre, Carleton University, Ottawa, ON K1A 0H3 Canada; 3 Department of Biology, Norwegian University of Science and Technology, 7491 Trondheim, Norway; 4 Department of Arctic Technology, The University Centre in Svalbard, PO Box 156, 9171 Longyearbyen, Norway; 5 Toxicology Laboratory, Department of Pharmacy, Faculty of Health and Medical Sciences, University of Copenhagen, Universitetsparken 2, 2100 Copenhagen, Denmark

**Keywords:** Contaminants, Greenland, Hg, Humans, Inuits, Mercury, Persistent organic pollutants, Polar bears, POPs, Seals, Sled dogs, Whales

## Abstract

Exposure to long-range transported industrial chemicals, climate change and diseases is posing a risk to the overall health and populations of Arctic wildlife. Since local communities are relying on the same marine food web as marine mammals in the Arctic, it requires a One Health approach to understand the holistic ecosystem health including that of humans. Here we collect and identify gaps in the current knowledge of health in the Arctic and present the veterinary perspective of One Health and ecosystem dynamics. The review shows that exposure to persistent organic pollutants (POPs) is having multiple organ-system effects across taxa, including impacts on neuroendocrine disruption, immune suppression and decreased bone density among others. Furthermore, the warming Arctic climate is suspected to influence abiotic and biotic long-range transport and exposure pathways of contaminants to the Arctic resulting in increases in POP exposure of both wildlife and human populations. Exposure to vector-borne diseases and zoonoses may increase as well through range expansion and introduction of invasive species. It will be important in the future to investigate the effects of these multiple stressors on wildlife and local people to better predict the individual-level health risks. It is within this framework that One Health approaches offer promising opportunities to survey and pinpoint environmental changes that have effects on wildlife and human health.

## Background

One Health aims to improve health and well-being through the prevention of risks and the mitigation of effects of crises that originate at the interface between humans, animals and their various environments (http://www.onehealthglobal.net). Given the close relationship between wildlife, sled dogs and humans in the North Atlantic, in combination with long-term biomonitoring programs using multiple veterinary tools over the last three decades, an Arctic One Health approach requires focus on few key species and study areas that interlink wildlife, human and ecosystem health. The present review will therefore discuss clinical and veterinary studies on polar bears (*Ursus maritimus*), ringed seals (*Pusa hispida*), narwhals (*Monodon monoceros*), killer whales (*Orcinus orca*), domestic sled dogs (*Canis familiaris*), and humans (*Homo sapiens*), and how shared information on these provide a base from which One Health can be operated. The focus will be on exposure of Arctic wildlife and Inuit populations to long-range transported contaminants and its interactions with climate change and infectious diseases (zoonoses).

### Search strategy

We conducted this literature review to give a systematic overview of the current knowledge on environmental contaminants and zoonosis in the Arctic and how it links to climate change in a multiple stressors framework. From our own working knowledge in the field, we identified “persistent organic pollutants (POPs)”, “mercury (Hg)” and “zoonoses” relevant for the Arctic and the link to “climate change” as overall themes/keywords. We used ScienceDirect, PubMed, Google, Google Scholar, ISI Web of Knowledge/Web of Science and Springer Link to locate peer-reviewed scientific articles and reports, using the thematic keywords “immune”, “endocrine”, “neuro”, “bone”, “diseases” and “multiple stressors” either alone or in combination. We also used dissertations and AMAP reports to obtain information from the grey literature. Although we attempted to be systematic within the scope of the review, we acknowledge that this is not an exhaustive representation of all the material that may exist in the scientific literature.

### The arctic environment as a sink for pollutants

Hunting and fishing have always been an important part of Arctic human existence in this harsh northern environment [1–6], and their intensity has increased with a growing Arctic human population bringing along more effective hunting methods, including larger and faster boats and fishing vessels, riffles and gillnets. Adding to these local stressors on Arctic ecosystems, anthropogenic pollution has become a ubiquitous problem that is particularly relevant in the Arctic environment [6–8]. In addition to elevated mercury (Hg), an array of anthropogenic chlorinated, brominated, and fluorinated persistent organic pollutants (POPs), all alien substances, have been introduced to the Arctic [1, 7, 9–14]. More specifically, the 1850s marked the period of early industrialization and was associated with elevated Hg emissions [10], whereas the onset of the 1940s marked the onset of large-volume worldwide use of industrial chemicals and pesticides. POPs and Hg typically originate from industrial and household emissions at temperate regions and are transported via global atmospheric and oceanic pathways that result in deposition in the Arctic environment [6, 7, 9, 11, 12, 15].

Arctic fish and wildlife rely on energy-rich fatty tissues as their main energy source in the harsh Arctic environment [16, 17]. Fatty tissues typically host various natural lipophilic compounds, such as specific vitamins (A and D) and endogenous steroid hormones transported by portal and periphery blood supply among others [8, 18–23]. However, most POPs and methyl-Hg, the bioavailable chemical from of Hg, are highly lipophilic as well and are therefore readily stored in lipid-rich tissues. Additional low excretion of these compounds results in a net intake of POPs and Hg over time, referred to as bioaccumulation, and is moreover transferred from prey to predator along the food chain resulting in biomagnification. As a result, apex predators such as polar bears, Arctic fox (*Vulpes lagopus*), seal spp., whale spp. and seabird spp., are exposed to the highest concentrations occurring the Arctic environment, already a major sink for POPs and Hg as described above. Finally, indigenous northerners and their dogs are ultimate sinks due to their traditional consumption of the aforementioned wildlife [7, 13, 14, 24, 25]. Newer perfluorinated compounds [poly- and perfluoralkyl substances (PFASs)], in particular perfluorooctane sulfonate (PFOS) and other long-chained poly- and perfluorocarboxylic acid (PFCAs) are proteinophilic and also biomagnify due to high resistance to biological degradation [7].

Long-range transported pollutants have been extensively monitored in the Arctic due to the high exposure of Inuit populations, resulting from their consumption of a marine diet consisting especially of apex marine predators high in contaminants [2, 3, 6]. Such biomonitoring activities have shown that, among these POPs, polychlorinated biphenyls (PCBs) continue to dominate and are of the greatest exposure concern, despite their ban decades ago [7]. However, other high-concentration POPs, amongst which organochlorine pesticides (OCPs), brominated flame retardants (BFRs), PFASs, and Hg can also be found at concentrations that raise concerns for the health of top predators and humans [7, 11, 26–28].

### Arctic wildlife and human health

POPs and Hg pose a health threat to Arctic top predators and humans because the compounds and their biotransformation metabolites have structural similarities to endogenous compounds. These anthropogenic compounds have been classified as endocrine disruptors or cellular toxicants acting via non-endocrine pathways, and thus negatively affect immune and neuro-endocrine functioning, growth and development, reproduction and general fitness [7, 8, 29]. Since the compounds potentially target different organ-tissues, the dietary exposure causes chronic and combined stress manifested through several health effects at the organism level [8, 30, 31].

There is evidence that high exposure poses a great risk to neonatal individuals during critical periods of development. Seasonal cycles of energy requirements for fasting, breeding, lactation, and migration lead to increased intake or catabolism of adipose tissue causing pulsed exposure to bioavailable contaminants circulating in the blood [32, 33]. In polar bears, for example, up to 70% of the total organochlorine body burden is transported from mother to offspring during lactation, resulting in cub adipose tissue concentrations that are approximately three times higher than those in their mothers [32, 34–37]. A female polar bears’ very first cubs are believed to be especially vulnerable since high contaminant exposure can affect normal development and growth [7, 8, 31]. In a meta-study, it has recently been modelled that chlorinated and brominated POPs, singularly or collectively, were far better predictors of declines in population densities in 14 polar bear subpopulations than were human population density, harvest rate and sea ice extention [38]. Indeed, circumarctic polar bear subpopulations are under influence of immunological, reproductive and carcinogenic consequences from POP exposure [27]. From a population conservation point of view, contaminants that reduce pregnancy, fecundity and survival in both males and females are among the most important to monitor in different Arctic subpopulations of polar bears, as well as other top predators and northerners relying on the same food web [39, 40].

East Greenland polar bears, killer whales, narwhals and ringed, harp (*Pagophilus groenlandicus*) and hooded seals (*Cystophora cristata*) carry very high concentrations of POPs and Hg [7, 11, 12, 15, 41]. Since Greenlanders in this region traditionally ingest significant quantities of adipose tissue from these species, they are among the Arctic people carrying the highest POP burdens [5, 6, 43, 44]. Such high exposure is likely to pose a health risk based on available literature on dioxin toxic equivalency factor (TEQ) and tolerable daily intake (TDI) guidelines [44, 45]. Human exposure to contaminants in Greenland has been evaluated from chemical analyses of prey species and food intake [5, 46, 47], showing that the TDI was exceeded for chlordane (CHL) by a factor of 3–6, while PCB exposure did not. However, none of these studies reported on polar bear and ringed seal blubber important to people’s exposure in East Greenland, where POP loads are known to be four times larger than in west Greenland. According to Nielsen et al. [48] it is recommended that Greenlanders reduce their exposure to PCBs and CHL by reducing their blubber intake. The Arctic Monitoring and Assessment Programme (AMAP) is therefore concerned about Arctic human health within a contaminant exposure context and their studies do support observations that Greenland hunters are particularly exposed to high PCB concentrations due to frequent ingestion of polar bear, killer whale, narwhal and seal tissues [6, 44, 49–51]. Studies from the Russian Arctic have shown that dioxin, furans and PCB exposure of neo- and prenatal children exceeded TEQ TDI levels by up to 33 times in the year 2000 [5, 52]. It has been shown that blood concentrations of PFASs including PFOS in male Inuits from East Greenland can be two to three times higher as compared to the Faroese population where local exposure has already been attributed with effects on the immune system [53–55].

Although certain PFASs have been associated with developmental and hormonal effects, immunotoxicity, and tumour growth in rodents [56], the impact of these compounds on human health appear to be inconclusive [56, 57]. Of the PCBs found in Greenlanders, the congeners CB-77, CB-126 and CB-169 attain a coplanar configuration similar to the very toxic dioxins and furans, and are in fact commonly found in Arctic wildlife [3, 5, 58]. These coplanar PCB congeners are characteristically highly potent inducers of aryl hydrocarbon hydroxylase activity [3, 5]. Furthermore, for Greenlanders, significant correlations were found between blood contaminant concentrations and calculated daily intake of POPs [51]. Hg exposure of Inuit people is also of great health concern and has been recognised as a neuro-endocrine and immune health problem in the societies of Faroe Islands, West Greenland (Avanersuaq, Thule) and Canada [6, 11, 59].

### Biomonitoring of wildlife health

Several of the environmental contaminants, such as PCBs and Hg are regulated by international agreements over the last 15 years through international treaties and conventions including the Stockholm Convention on POPs (http://chm.pops.int) and Minamata Convention on Hg (http://www.mercuryconvention.org). However, over the last two decades, the concentrations of the highly toxic PCBs, chlordane pesticides and Hg have remained essentially unchanged or even increased in polar bears inhabiting contaminant hot spots, such as Greenland and Hudson Bay [10, 12–14, 60, 61]. This is likely due to climate change effects on food web interactions, generational transfer, and continued secondary and unintentional emissions [12, 13, 17, 62]. For Hg, body burdens even appear to be continuously increasing in most top predators in the central Arctic reaching up to 20-fold baseline levels of pre-industrialisation [10, 11, 60, 63].

For three decades the AMAP program and associated subprograms have therefore monitored the health of Arctic wildlife and humans [1, 3, 5–7, 41, 60]. For the purpose of studying contaminant concentrations, spatial and temporal trends, and human exposure, these programs have used ringed seals and polar bears as key monitoring species [7, 64]. From the East Greenland region, a large number of organ–tissue samples from polar bears have been obtained since the 1980s as part of the traditional hunt [12, 42]. Likewise, from Svalbard, Canada and Alaska, adipose tissue and blood has been archived, facilitating the spatial and temporal trends which are further supported by similar samples from ringed seals [7]. With respect to East Greenland polar bears, histopathological, gross morphological and bone composition and morphological investigations have been carried out since 1999 [8, 31, 65, 66]. These studies have provided a unique opportunity to investigate the potential organ-specific effects of POP exposure [8]. Similar to polar bears, analyses of bone density and histopathology have been carried out on West Greenland ringed seal populations [67, 68] and on Alaska polar bears, ringed seals and whale spp. [69–72].

Interpretations and conclusions in wildlife health studies linked to contaminant exposure are typically based on correlational and descriptive interpretations, unavoidably confounded by a plethora of factors affecting the physiological state of a free-ranging animal. To improve the understanding of the effects of exposure to real-world contaminant cocktails experimental exposure studies have been performed on sled dogs and domesticated Arctic foxes, being possible surrogate model species for *Canidae* spp. In captive sled dogs and Arctic fox studies, which both included a cohort fed a naturally POP contaminated diet of minke whale (*Balaenoptera acutorostrata*) blubber, it has been possible to define and compare POP exposed and unexposed reference groups in direct relation to an array of effects such as on reproductive organs and other internal organs, the skeletal system, immune and endocrine systems, and POP dietary accumulation, biotransformation and toxicokinetics [7, 8, 31].

### Biological effects

**Fig. 1:**
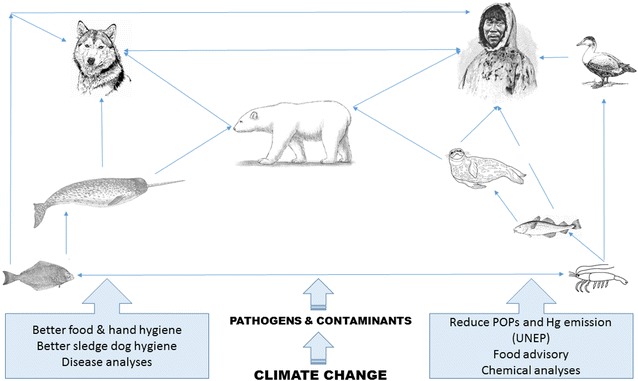
A simplification of the marine food web and One Health in the Arctic. Climate change affect the presence of pathogens and composition of contaminants in Arctic wildlife that is handled and eaten by Inuits and sled dogs transferring contaminants and pathogens into the local societies. Inuits and sled dogs share microbiomes which increases the risk of zoonotic infections. Actions to be taken is given in callouts

In the following sections we review the available literature of One Health in the Arctic (Fig. 1). We present and discuss the results from empirical studies of wildlife and compare these with controlled studies of sledge dogs and arctic foxes in the context of One Health and health effects in humans. The following sections are divided into first specific organ-systems and after that the relatively sparse information on multiple effects health effects are presented and discussed.

#### Chronic diseases

Chronic diseases, including diabetes, upper respiratory and recurrent middle ear infections, cancer, and osteoporosis, are becoming an epidemic in the Arctic and may be partially caused by chronic POP exposure and associated endocrine disruption [3, 5, 73]. It is apparent that subsistence hunters in East Greenland are exposed to mixtures of POPs that include both toxic parent compounds and also their derived metabolites [74]. Some POPs can induce (hepatic) cytochrome (CYP) 450 enzymes in Arctic people that may interfere with normal metabolic processes and homeostasis of various intrinsic hormones and vitamins influencing immunity and reproduction [1, 3, 5, 75, 76]. Likewise, Sandau et al. [77] found that metabolism of PCBs was significantly negatively correlated with thyroid hormones, namely free thyroxine, in northern peoples in the Ungava region in northern Quebec, Canada. These findings of associations between POP exposure, induction of CYP-450 enzymes, and changes in thyroid hormone concentrations, may play a role in the development of chronic diseases [5].

#### Bone mineral density

Studies of polar bears suggest that osteoporosis may be a problem for especially the male bears that do not have the evolutionary physiological mechanisms that females have, putting them at high risk of POP related bone mineral density declines [8, 66, 78]. The studies of polar bears have shown reductions in bone mineral density due to PCB exposure all supported by studies on seals in the Baltic Sea and alligators in Florida, USA [79–81].

A single study has been published on osteoporosis in relation to POP exposure in Greenlanders [82]. The study focused on quantitative ultrasound parameters (QUS) at the right calcaneus of 153 people from Southwest Greenland and found blood plasma CB-153 concentrations strongly and negatively associated with the three QUS parameters. While the relationship was no longer significant when normalizing for age and weight, people in Southwest Greenland belong to some of the lowest POP exposed Greenlanders and effects on bone composition are therefore not likely to occur [5, 6]. Another study of Cree women from Eastern James Bay in Canada showed that bone stiffness index was negatively associated to blood concentrations of CB-105 and CB-118 [83].

#### Endocrine disruption

Endocrine disruption from POP exposure is likely to be a challenge in Arctic wildlife [30, 84]. Polar bears have been in focus over the last decades and both steroid hormones and thyroid hormones seem to be impacted by POP parent compounds and their metabolites, mimicking hormone activity in both the transport pathways and receptor mechanisms and affecting overall health and survival [8, 30]. These investigations have been backed up by controlled experiments on sled dogs and Arctic foxes [8, 84]. Likewise, in Inuit peoples, disruption by POPs of the thyroid and steroid hormone endocrine axis is influencing physiological endpoints with effects on reproduction, cancer, and immunity [6, 85].

Studies on polar bears from Svalbard indicate that plasma steroid levels may be affected by POP exposure, particularly PCBs and their hydroxylated metabolites (OH-PCBs). In females, pregnenolone and androstenedione in the blood appeared to be significantly negatively correlated to several hydroxylated PCBs [86], indicating that these POPs may affect early or perhaps rate-limiting steps in steroidogenesis. The data also indicated that CYP-17 may be a target for OH-PCBs and consequently the reproductive potential of female polar bears. PCBs also appear to affect male plasma steroids, in particular androgen levels in Svalbard polar bears [87]. Concentrations of the most masculinizing steroids, dihydrotestosterone and testosterone, were negatively correlated to levels of a large number of PCB congeners.

#### Reproductive organs

Studying reproductive organs in wildlife is often based on necropsy samples as clinical investigations are rather difficult. Necropsy of human reproductive organs from Arctic populations has for ethical reasons not been studied, though the investigation of endocrinology and reproductive organs in wildlife and domesticated species (sled dogs and Arctic fox) can give some important information. For example, the presence of orchitis in polar bear testes [88] may be of importance while studying testicular dysgenesis syndrome in Inuits. Seasonal hormone and semen production in Arctic fox was found to be affected by POPs [89, 90]. Similarly, there are indications that the size of polar bear penis bone, testicles and ovaries may be inversely correlated to PCB concentrations [66, 88]. Such information may be of use in human medicine when autopsies are not an option and when POPs are suspected of having endocrine disruptive effects in humans.

#### Immune effects

Environmental contaminants have been shown to modulate all measurable aspects of cellular and humoral immunity in marine mammals [89]. Resistance against acute and chronic diseases also depends on the optimal function of the immune system [91, 92]. For years, POPs have been known to modulate immunity, and while the mechanisms of immune suppression are not fully understood, it includes both humoral cell-mediated systems [3, 92–95]. Several studies have reported immune effects in Arctic wildlife, including polar bears, ringed seals, sled dogs and Arctic foxes [8], demonstrating that contaminants are reaching levels that can cause significant changes in physiology and immune fitness, with important consequences for resistance to disease.

Polar bear immunity was assessed in a comprehensive study in Svalbard and Hudson Bay, and negative associations were found between PCB exposure and serum immunoglobulin G (IgG) levels, antibody titres against influenza virus and reovirus following immunizations, and lymphocyte proliferation [96, 97]. In a controlled study on Greenland sled dogs, exposed pups, but not adults, showed reduced and delayed IgG antibody production with circulating levels of IgG in all pups correlating to blood concentrations of several POPs [8]. Hepatic mRNA expression of interleukin-1β (IL-1β), an important pro-inflammatory cytokine, in ringed seals from Svalbard were positively correlated with hepatic POP levels [98]. Similarly, hepatic IL-1 mRNA expression was positively correlated with blubber PCB burdens in 41 ringed seals in northern Labrador, Canada [99]. These studies illustrate that in vivo real life exposure to contaminants cause measurable changes in immune function in Arctic wildlife, and thus likely humans.

In vitro experiments, where immune cells are exposed to contaminants under laboratory conditions in order to characterise effect levels, have also been carried-out in Arctic wildlife species. East Greenland ringed seal leukocytes were exposed to four PCB congeners (CB-138, -153, -169, and -180) and two PFASs, i.e. PFOS and perfluorooctanoic acid (PFOA), and it was found that PCBs but not PFASs cause significant suppression of lymphocyte proliferation at relevant environmental concentrations for seals [100]. Beluga whale (*Delphinapterus leucas*) leukocytes were also used to show that low levels of Hg exposure can cause significant reductions in lymphocyte proliferation and intracellular thiol production, and significant induction of metallothionein [101]. Altogether, studies of free-ranging animals and in vitro experiments suggest high contaminant loads in the Arctic can induce immune suppression which affect the ability to respond to intruding infectious pathogens as previously suggested for marine mammals [7, 8, 31, 89] and Inuit and other northern peoples [6]. For example, prenatal exposure to PCBs has been suspected to play a role in the relatively high incidence of acute respiratory infections and middle ear inflammation in Inuit children [6, 102–106].

#### Neurological effects

Multiple environmental contaminants can cross the blood–brain barrier and exert effects on the brain functioning thus causing neurobehavioral effects. Through various mechanisms, contaminants can influence mating and other reproductive behaviours, motivation, communication, aggression, dominance and other social behaviours, as well as learning and other cognitive abilities [29, 107]. Field studies of behavioural effects of POPs in polar bears are difficult to conduct due to logistical and ethical constraints. However, several POPs reported in brain tissue of polar bears [74, 108–112] are confirmed or suspected developmental neurotoxicants in humans and experimental animals [29, 107, 113, 114]. POPs might affect brain function or development through many mechanisms, for instance by interacting with brain neurotransmitter systems [113]. Also, the ability of some POPs to induce epigenetic changes could present a mechanistic pathway of neurodevelopmental perturbations [115–117]. Associations between neurochemical and epigenetic biomarkers and Hg levels in brain tissue have been reported for polar bears [11, 118, 119]. Despite relatively low concentrations of Hg, significant negative correlations were found between both Hg concentrations and *N*-methyl-d-aspartic acid (NMDA) glutamate receptors. NMDA glutamate and genomic methylation is important for animal health, behaviour, reproduction, and survival, and their reduction may have population-level effects for polar bears [120].

Another proposed mechanism of developmental neurotoxicity is through thyroid hormone disruption; thyroid hormones are essential for proper neurodevelopment of the foetus and early neonate [121, 122]. Thus, the high levels of POPs reported in 4-month old polar bear cubs [34] and their associated thyroid disrupting properties in cubs [123, 124] raises concern for neurodevelopmental effects in polar bears. The relatively high concentrations of several POPs reported in polar bear brains may cause adverse effects, with a possible heightened susceptibility during the more sensitive foetal and neonatal stages of brain development [29, 125]. Similarly to humans, this could alter behavioural traits and reduce cognitive abilities related to memory and learning in offspring. For polar bears, this could functionally reduce hunting skills or alter mating behaviour, and thus ultimately affect reproduction and survival.

### Multiple stressors

In the previous section we discussed the important risk posed by environmental contaminants in Arctic wildlife and human populations for a wide range of physiological health endpoints. Arctic ecosystems, however, are being stressed by more than contaminants, resulting in a situation of multiple cumulative stress for wildlife and humans. Two major additional aspects to consider in the study of Arctic health is climate change and infectious diseases. Climate change has a duel impact, acting through alteration of food web pathways for contaminants [61, 126] and spreading and virulence of zoonotic diseases associated to sea ice conditions [127–129]. A pollution induced increase in disease rates due to immunotoxic effects of POPs and Hg can increase the likelihood and risk of disease transfer from animals to humans (zoonoses) as the proportion of infected Arctic wildlife increases [30, 89, 130–132]. A large volume of marine and terrestrial wildlife is consumed by humans in the Arctic, often raw and inadequately frozen, and this likely increases the risk of zoonotic diseases [8].

#### Climate change and contaminant exposure

Arctic wildlife have received considerable focus as they, depending on the regional subpopulation, are threatened most dramatically by climate change due to observed and projected loss of sea ice, which has important implications for ice-associated hunters like polar bears [133–135]. Modeling has shown that southernmost polar bear subpopulations in the Hudson Bay are at greatest risk, and will struggle to persist throughout this century [136]. In fact, models have also predicted two-thirds of the world’s polar bears could disappear if greenhouse gas emissions continue to increase as predicted [134, 137]. This has been linked to the occupation of large home range sizes and the requirement of higher energetic costs and thus higher feeding rates, which can lead to increasing blood PCB concentrations [138, 139]. In some regions, the decline of sea ice extent has resulted in changes in the presence of seal species that polar bears prey upon, and this has been shown to cause increased bioaccumulation of certain POPs as more contaminated prey are being consumed [61, 140]. Dietary shift towards feeding on plants, berries and caribou (*Rangifer tarandus*) and seabird eggs [141–143] will most likely decrease and increase, respectively, the exposure to POPs. Furthermore, climate warming induced migration of warm water adapted fish species [144, 145] may act as bio-vectors increasing contaminant levels in marine Arctic ecosystems [146, 147], ultimately causing increased bioaccumulation and biomagnification of these compounds to humans and other high trophic marine wildlife [30, 126]. McDonald et al. [147] conducted a review on ecological impacts of global climate change on POP and Hg pathways and exposures in arctic marine ecosystems, and documented that lower sea ice extent mediated dietary changes were associated with higher contaminant levels in some populations of polar bears, ringed seals, and thick-billed murres (*Uria lomvia*), but the influence of changing trophic interactions on POP levels and trends varied widely in both magnitude and direction.

#### Climate change and infectious diseases

Climate change not only threatens to alter contaminant dynamics by changing Arctic ecosystems, but these same factors influencing the presence and extent of different species in the Arctic will have implications for the introduction of novel infectious diseases to the region. Climate change has been deemed the most important factor in the emergence of infectious diseases, and nowhere else in the world is climate change occurring as fast as in the polar regions [148, 149]. A warming climate may profoundly affect disease dynamics in the Arctic by changing the species composition and northward invasion of disease vectors and transport of pathogens [149, 150]. In addition, increased survival of infected animals during milder winters may further increase the risk of pathogen reservoir in marine mammals including that of zoonosis [149, 150]. Moreover and as discussed above immunotoxic contaminants may increase disease-related mortality and morbidity of Arctic marine mammals [129].

### Perspectives and recommendations

Further efforts are required to understand the toxicokinetics and toxicodynamics of POPs and Hg in Greenland wildlife and peoples in this changing Arctic in order to better predict the individual-level health risks associated with contaminant exposure. Arctic top predators are sentinels for humans as they consume the same diet and act as potential vectors for zoonotic transfer to humans due to harvesting. Combining correlational studies on wildlife health with experimental work on surrogate species, such as the sled dog, will allow better understanding of the proximate toxic pathways behind exposure to contaminants and infectious diseases, their interactions, and the driving role of a rapidly changing climate. Doing this offers a promising One Health approach to survey and pinpoint environmental change and multiple stressors that may have effects on wildlife and human health [7, 8, 31, 151–153]. The warming Arctic climate is suspected to influence abiotic and biotic long-range transport and exposure pathways of contaminants to the Arctic. As a result there will be likely increases in POP exposure of Arctic wildlife and human populations, while exposure to vector-borne diseases and zoonoses may increase as well through range expansion and introduction from invasive species. Broad, and nevertheless in-depth studies on the occurrence and human health risk of Arctic zoonoses, and their interactive effects with climate change and contaminant exposure are pending, as well as an increased effort to educate the relevant groups of the public regarding safe handling of wildlife.

## Abbreviations

AMAP: arctic monitoring and assessment program
BFRs: brominated flame retardants
BMD: bone mineral density
CHL: chlordane
Hg: mercury
IL: interleukin
IgG: immunoglobulin G
NMDA: *N*-methyl-d-aspartic acid
OH-PCBs: hydroxylated PCBs
OCPs: organochlorine pesticides
PCBs: polychlorinated biphenyls
PFASs: poly- and perfluoralkyl substances
PFOS: perfluorooctane sulfonate
PFCAs: poly- and perfluorocarboxylic acid
POPs: persistent organic pollutants
QUS: quantitative ultrasound parameters
TEQ: dioxin toxic equivalency factor
TDI: tolerable daily intake
